# DSCnet: detection of drug and alcohol addiction mechanisms based on multi-angle feature learning from the hybrid representation of EEG

**DOI:** 10.3389/fnins.2025.1607248

**Published:** 2025-06-18

**Authors:** Jing Wu, Nan Zhang, Qilei Ye, Xiaorui Zheng, Minmin Shao, Xian Chen, Hui Huang

**Affiliations:** ^1^College of Computer Science and Artificial Intelligence, Wenzhou University, Wenzhou, China; ^2^Data Resources Division, Wenzhou Data Bureau, Wenzhou, China; ^3^Department of Drug Rehabilitation and Correction, Wenzhou City Huanglong Compulsory Isolation Drug Rehabilitation Center, Wenzhou, China; ^4^Department of Otolaryngology, Wenzhou Central Hospital, Wenzhou, China; ^5^Information Technology Center, Wenzhou Polytechnic, Wenzhou, China

**Keywords:** electroencephalograms, alcoholism, drug addiction, computer-aided diagnosis, convolutional neural networks, classification

## Abstract

**Introduction:**

Drug and alcohol addiction impair neurotransmitter systems, leading to severe physiological, psychological, and social issues. Electroencephalography (EEG) is commonly used to analyze addiction mechanisms, but traditional feature extraction methods such as time-frequency analysis, Principal Component Analysis (PCA), and Independent Component Analysis (ICA) fail to capture complex relationships between variables.

**Methods:**

This paper proposes DSCnet, a novel neural network model for addiction detection. DSCnet combines embedding layers, skip connections, depthwise separable convolution, and our self-designed Directional Adaptive Feature Modulation (DAFM) module. DAFM is a key innovation that adaptively adjusts feature directionality, extracting global features from EEG signals while preserving spatiotemporal information. This enables the model to capture neural activity patterns related to addiction mechanisms. DSCnet uses a multi-angle feature extraction strategy, emphasizing information from various perspectives.

**Results:**

On the drug addiction dataset, DSCnet achieved 85.11% accuracy, 85.13% precision, 85.12% recall, and 85.12% F1-score. On the UCI alcohol addiction dataset, it achieved 84.56% accuracy, 84.73% precision, 84.56% recall, and 84.63% F1-score.

**Discussion:**

These results outperform existing models and demonstrate a balanced performance across both datasets, highlighting DSCnet's potential in addiction detection.

## 1 Introduction

### 1.1 Motivation

Drug addiction and Alcohol Use Disorder (AUD) are complex, chronic brain diseases. Despite involving different substances, their addiction mechanisms share many similarities. Drug addiction leads to changes in neurotransmitter levels, particularly dopamine, which profoundly affect the brain's reward system (Koob et al., 2023; Raji et al., 2025). The use of drugs disrupts normal dopamine release and reuptake processes, resulting in heightened cravings and difficulty experiencing pleasure from non-drug-related activities (Volkow, 2024). Similarly, AUD is characterized by compulsive drinking, loss of control, and negative emotions when alcohol is unavailable. Symptoms include repeated urges to drink, increased consumption, and persistent heavy drinking to avoid withdrawal symptoms. Long-term alcohol abuse not only causes physical health issues, such as liver damage and cardiovascular diseases, but also negatively impacts mental health, leading to depression and anxiety (Koob et al., 2020). Additionally, alcohol misuse often results in social dysfunction, including reduced work performance, family conflicts, and social isolation (Koob, 2024). Both drug addiction and AUD severely affect neurotransmitter systems, resulting in functional impairments and deteriorating psychological states in affected individuals.

Therefore, studying the mechanisms of addiction and strategies for its inhibition is a very important research direction. Previous studies have investigated the effects of resveratrol (Yunusoğlu, 2021) and linalool (Yunusoğlu, 2022) on alcohol-induced conditioned place preference (CPP) in mice, demonstrating the potential of both substances in reducing alcohol dependence behaviors. Meanwhile, addiction research in humans has mainly relied on diagnostic scales, such as the Addiction Severity Index (ASI) (Ljungvall et al., 2020; Rodriguez et al., 2023; Schawo et al., 2024), the Diagnostic and Statistical Manual of Mental Disorders (DSM) (Ersche et al., 2012; Yang et al., 2023), and the International Classification of Diseases (ICD) (Saunders, 2017). However, these scales mainly focus on psychological aspects and lack reliable physiological and behavioral indicators, making them susceptible to human factors. Thus, accurately assessing addiction severity and determining whether a patient remains addicted is crucial for recovery, directly influencing treatment plan formulation, adjustment, and implementation.

To address the shortcomings of traditional scales, modern research has increasingly applied electroencephalography (EEG) (Soufineyestani et al., 2020) and event-related potentials (ERP) (Fathi et al., 2024) to explore the neural mechanisms that differentiate healthy individuals from those with brain disorders. These techniques have demonstrated effectiveness in diagnosing various conditions, including epilepsy (Xin et al., 2022), Alzheimer's disease (Vicchietti et al., 2023), alcohol addiction (Farsi et al., 2020), gaming addiction (Pangistu and Azhari, 2021), and drug addiction (Zeng et al., 2022). Utilizing these physiological indicators allows for a more comprehensive and accurate assessment of addiction, enhancing diagnostic intelligence and treatment effectiveness.

EEG is a key diagnostic tool in addiction, providing real-time monitoring of brain activity. It offers insights into neural states related to withdrawal and cravings, helping clinicians understand addiction's underlying mechanisms. The non-invasive, cost-effective nature of EEG allows repeated measurements, ideal for tracking changes in brain activity during addiction. This helps assess treatment progress and tailor interventions to individual needs. As addiction treatment moves toward personalized medicine, integrating EEG into diagnosis and therapy is crucial. It decodes the neural processes behind addiction and aids in developing targeted, effective treatment strategies tailored to each patient.

### 1.2 Related works

In previous studies, low-dimensional representations of EEG signals have typically been constructed using time-frequency analysis, Principal Component Analysis (PCA), and Independent Component Analysis (ICA), with machine learning or deep learning models used for classification. For example, Subasi and Gursoy (2010) used Discrete Wavelet Transform (DWT) to decompose EEG signals into different frequency bands, followed by PCA, ICA, and Linear Discriminant Analysis (LDA) for dimensionality reduction, and classified the extracted features using Support Vector Machine (SVM). Meynaghizadeh-Zargar et al. (2023) applied the High-Comparative Time Series Analysis (HCTSA) method to extract features from EEG signals, using Logistic Regression (LR), SVM, and Random Forest (RF) for classification. Farsi et al. (2020) performed feature extraction using PCA and applied Artificial Neural Networks (ANN) for classification. Anuragi and Sisodia (2019) used Flexible Analytic Wavelet Transform (FAWT) to decompose EEG signals and train models such as SVM and Naive Bayes for classification. Shen et al. (2023) classified alcoholic EEG signals using whole-brain connectivity analysis and deep learning, employing mutual information algorithms and Continuous Wavelet Transform (CWT), along with 2D and 3D Convolutional Neural Networks (CNNs). Liang et al. (2024) proposed a CNN-based model for classifying sleep spindles and applied transfer learning to transfer features from healthy subjects to insomniac subjects, achieving effective classification results. Pain et al. (2023) integrated alcohol-related EEG electrode features with inherent connectivity patterns from spatially distributed electrodes, representing these as graphs and classifying the resulting alcoholic and non-alcoholic graphs using Graph Neural Networks (GNNs), validated with a Phase Lag Index (PLI) connectivity estimator and Graph Convolutional Networks (GCNs).

However, single-perspective EEG feature extraction methods struggle to capture the complexity and diversity of the signals. For instance, the RMRE model proposed by Huang et al. (2024), which uses low-rank constraints and structural signal regularization, still faces challenges in capturing the global structure of EEG signals as complexity increases.

Moreover, EEG-based addiction diagnosis studies often involve small datasets, with fewer than 10 subjects per group of addicts and healthy controls, limiting the generalizability of the models. These studies typically focus on the classification and diagnosis of a single type of addiction, such as drug or alcohol addiction.

### 1.3 Objective and contributions

The objective of this study is to leverage EEG signals to differentiate between healthy individuals and those with substance addictions, specifically drug and alcohol addiction. By harnessing the power of deep learning–particularly through a hybrid EEG representation and advanced feature extraction techniques–we aim to improve the accuracy of addiction classification while gaining deeper insights into the neural abnormalities associated with addiction. This approach could support early diagnosis and contribute to more precise, targeted treatment strategies.

The contributions of this paper are summarized as follows:

- We employed an embedding layer from deep learning to construct low-dimensional representations of EEG, moving beyond traditional methods like time-frequency analysis, PCA, and ICA. To avoid the potential loss of critical features, we introduced skip connections to form a hybrid representation of EEG, preserving important information.
- In DSCnet, we integrate depthwise separable convolution for local feature extraction and our self-designed Directional Adaptive Feature Modulation (DAFM) module for global feature extraction. Additionally, we incorporate the CoTAttention module to capture both dynamic and static features, enabling comprehensive mixed feature extraction from EEG signals.
- We collected resting-state EEG data from 60 drug addicts and 70 healthy individuals. After screening, we constructed a refined dataset consisting of 46 drug addicts and 54 healthy subjects to rigorously validate the model's effectiveness.
- We tested DSCnet on the UCI alcohol addiction dataset, demonstrating that our model not only excels in drug addiction classification but also outperforms previous models in alcohol addiction classification tasks.

### 1.4 Paper organization

The organization of the paper is as follows. Section 2 presents the materials, which are divided into three subsections: Drug Addiction Dataset, Alcohol Addiction Dataset, and Data Standardization. Section 3 introduces the proposed DSCnet model, outlining its three key stages: Hybrid Representation of EEG, Multi-angle EEG Representation Learning, and Classifier. This section also concludes with a discussion of the innovations integrated into the DSCnet model. Section 4 is dedicated to the experiments and results, further divided into two subsections: Comparison with Existing Algorithms and Ablation Study. Section 5 discusses the strengths and limitations of DSCnet, its future potential, and extensions to other brain disorders. Finally, Section 6 offers the conclusion.

## 2 Materials

In this paper, we utilize two datasets for our study. The first is a drug addiction dataset that we collected and meticulously processed to ensure data quality and consistency. The second is an alcohol addiction dataset obtained from the UCI Machine Learning Repository, which has been widely used in previous research. The following sections provide a comprehensive introduction to both datasets, including their sources, data collection procedures, and key characteristics.

### 2.1 Dataset on drug addiction

In this experiment, we constructed a drug addiction dataset consisting of 60 participants who used either novel or traditional drugs, along with 70 healthy controls. The exclusion criteria included: a history of mental illness or current mental disorders; severe and unstable physical illnesses; inability to complete questionnaires and assessments; severe suicidal tendencies; epilepsy or other neurological diseases that could trigger random electroencephalographic activity; and individuals with contraindications for electroencephalography (EEG) or event-related potential (ERP) testing. The experimental process and data processing methods refer to Meynaghizadeh-Zargar et al. (2023). In this study, EEG signals were recorded under controlled conditions. The experiment was conducted in a standard laboratory environment with low ambient noise to minimize external interference. The lighting in the room was standard, using natural light or regular artificial light, ensuring no additional visual or light stimuli were applied to the participants. During the EEG recording, participants followed a 10-min protocol: 4 min with eyes closed, 2 min with eyes open, and another 4 min with eyes closed. No external sensory input was applied during the entire procedure to ensure that the EEG signals reflected natural variations. Additionally, each participant signed an informed consent form regarding the experiment.

Ultimately, after screening, we retained data from 54 healthy controls and 46 drug addicts. Each subject's data is stored in two MAT files.

**Figure 1:**
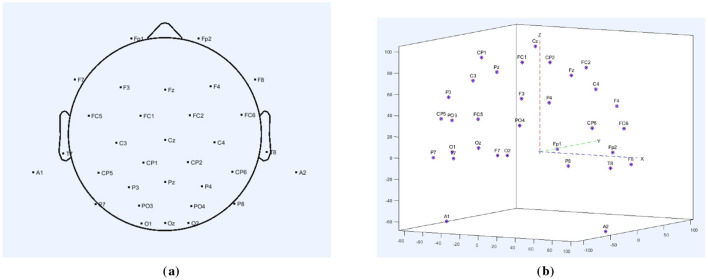
**(a)** 32-channel electrodes' 2D distribution map. **(b)** 32-channel electrodes' 3D distribution map.

This study employed a 32-channel electrode array for EEG signal acquisition, with the electrode distribution shown in Figure 1. Figure 1a presents the 2D distribution of the 32 channels, while Figure 1b shows the 3D distribution. The electrode array was connected to an EEG amplifier to enhance the signals. The collected EEG signals were recorded at a sampling rate of 1,000 Hz and stored using the Neuracle system.

For data processing, we used Matlab to analyze the EEG data of all subjects, following these specific steps:

Step 1: We determined the electrode channel locations to ensure accurate spatial mapping of the EEG data for subsequent analysis.
Step 2: We applied a 50 Hz notch filter to eliminate power line interference, which helped remove electrical noise caused by the power supply and improved the signal-to-noise ratio.
Step 3: A 0.5–64 Hz bandpass filter was used to retain the most relevant frequency range for EEG analysis while eliminating low-frequency drift and high-frequency noise.
Step 4: The data was downsampled to 128 Hz to reduce the data size for efficient processing while preserving important information.
Step 5: We extracted time segments related to eyes-closed events to focus on the relevant brain activity and reduce the impact of external distractions.
Step 6: Independent Component Analysis (ICA) was performed to remove eye movement artifacts and other unwanted artifacts, enhancing the purity of the EEG signal.
Step 7: Finally, we re-referenced the data using electrodes A1 and A2 to improve signal quality by reducing common noise and standardizing the data for analysis.

### 2.2 Dataset on alcohol addiction

The UCI dataset (Zhang et al., 1995), provided by Henri Begleiter at the Neurodynamics Laboratory of the State University of New York Health Center in Brooklyn, includes two groups of subjects: alcoholics and controls. The study involved 122 subjects, each completing 120 trials with different stimuli. In each trial, a subject was presented with either a single stimulus (S1) or two stimuli (S1 and S2). When two stimuli were presented, there were two conditions: a matched condition, where S1 was identical to S2, and a non-matched condition, where S1 differed from S2.

There are three versions of the EEG dataset: the Small Data Set, which includes data for two subjects; the Large Data Set, comprising data for 10 alcoholics and 10 controls; and the Full Data Set, which is intended to contain data from 120 trials for 122 subjects, although some data are missing. We selected the Full Data Set, and after removing files with format errors, empty files, and trials marked with “err,” we ended up with data from 122 subjects, totaling 10,880 trials. According to the original description of the alcohol addiction dataset, we found that a 61-electrode cap was used during data collection. Therefore, we removed the electrode data labeled as X, Y, and nd from the dataset, and each sample finally contains data from 61 channels.

### 2.3 Data standardization

Both datasets in this study utilized the same data normalization method. For each EEG segment *X*, the mean and standard deviation across all channels were calculated for each time point, resulting in corresponding values for each time point. Next, the global mean and standard deviation for all samples in the entire dataset were computed, yielding the overall mean μ and standard deviation σ for each time point. Based on these global statistics, mean normalization was performed on each sample. The formula for normalization is as follows:


(1) $$\begin{array}{l} f\left(X\right)=\frac{X-\mu }{\sigma } \end{array}$$

## 3 Methodology

**Figure 2:**
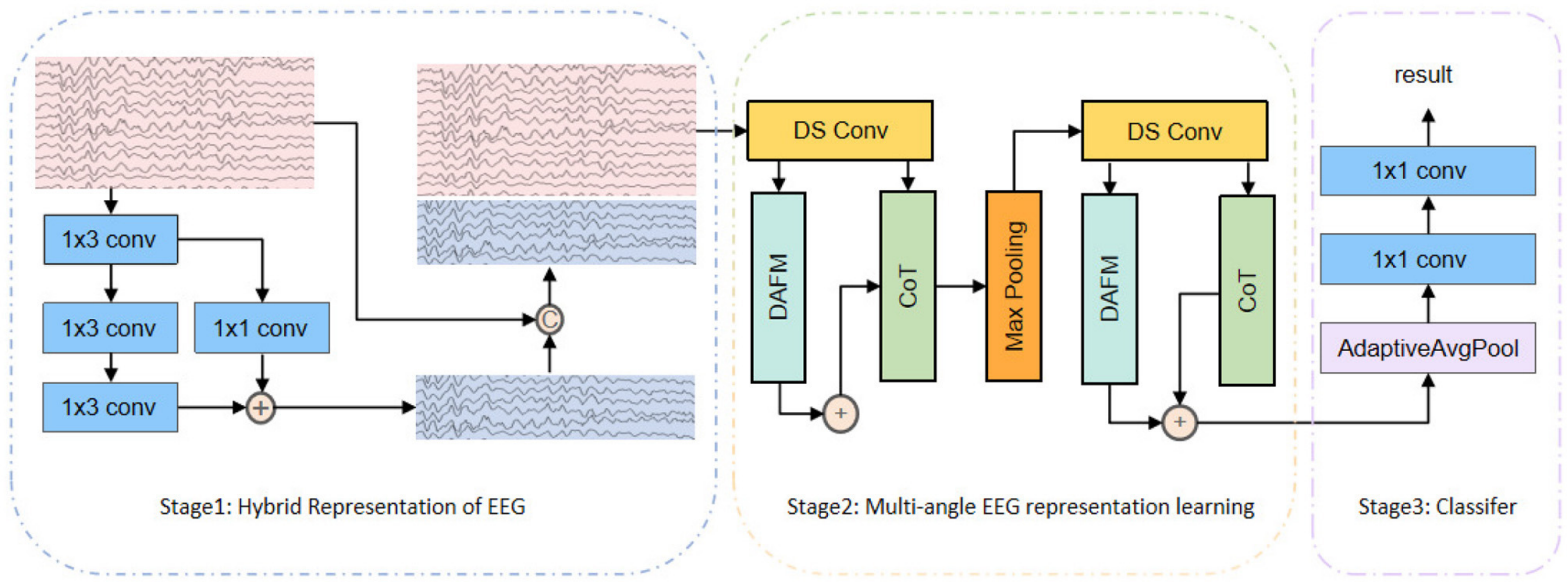
The overall structure of DSCnet consists of three stages: Hybrid Representation of EEG, Multi-angle EEG Representation Learning, and Classifier. In Stage 2, it incorporates three modules: depthwise separable convolutions for a local perspective, Directional Adaptive Feature Modulation (DAFM) for a global perspective, and the CoTAttention module for both dynamic and static perspectives.

As shown in Figure 2, the proposed DSCnet framework consists of three stages: Hybrid Representation of EEG, Multi-angle EEG representation Learning, and Classifier. In Stage 2, there are three modules: depthwise separable convolutions for local perspective, Directional Adaptive Feature Modulation (DAFM) for global perspective, and the CoTAttention module for dynamic and static perspectives. This section will provide a detailed description of these structures and their roles within the model.

### 3.1 Stage 1: hybrid representation of EEG

Raw EEG data typically exhibit high dimensionality and contain substantial noise and redundant information. Previous methods often employed Principal Component Analysis (PCA), Linear Discriminant Analysis (LDA), frequency domain analysis, and time-frequency analysis to construct low-dimensional representations of EEG, effectively removing this noise and redundancy. In this approach, we utilized an embedding layer to capture various features and patterns in the EEG signal through multiple convolutional operations, resulting in a low-dimensional representation of the EEG.

As shown in Figure 2, the embedding layer consists of three 1 × 3 convolutional layers and a residual block that includes a 1 × 1 convolution. After processing the raw EEG signal, which has a dimension of *C* ×1 × *T*, through these layers, we obtain a compressed representation. The skip connections from the residual block allow the input signal to be directly passed to subsequent layers, alleviating the vanishing gradient problem and accelerating the model's convergence, thus enhancing expressiveness and stability.

Ultimately, by connecting along the channel dimension, we form a Hybrid Representation of EEG, which serves as input for the next stage. Compared to previous methods, this approach provides a multi-dimensional representation of the EEG. The embedding layer captures the spatiotemporal features in the EEG signal and compresses the semantic information represented by different events, aiding in the extraction of richer and more discriminative features, thereby improving the effectiveness of subsequent analyses.

### 3.2 Stage 2: multi-angle EEG representation learning

In EEG analysis, learning features from multiple perspectives helps capture diverse information. As shown in Figure 2, we performed multi-round, multi-angle feature learning by repeatedly extracting information from different angles of the EEG signal to achieve comprehensive feature learning. The Hybrid Representation of EEG generated in the first stage undergoes the following processing steps:

First, depthwise separable convolution is applied to extract local features. Then, these features are further extracted by the CoTAttention module and the DAFM. Finally, the resulting features are summed element-wise and enhanced by max pooling to retain the most significant features.

#### 3.2.1 Depthwise separable convolution for local perspective

To capture local spatiotemporal information in EEG, we employ depthwise separable convolution for feature extraction from the Hybrid Representation of EEG. Unlike standard convolution, depthwise separable convolution is composed of two parts: depthwise convolution and pointwise convolution.

First, depthwise convolution applies convolution operations separately on each channel, focusing on capturing detailed information within the local region of each electrode. This method efficiently extracts spatial neighborhood activity patterns for each electrode, making it particularly effective in detecting local features in specific electrode regions. Additionally, depthwise convolution can also capture variations and patterns over time, identifying short-term neural activity changes and transient EEG wave features, as the convolution kernel slides along the time axis.

Second, pointwise convolution performs a 1 × 1 convolution operation, integrating the local information across different channels, thereby capturing inter-channel correlations.

This approach allows us to extract detailed local spatiotemporal features from the Hybrid Representation of EEG, enhancing the sensitivity to important signal patterns.

#### 3.2.2 DAFM from a global perspective

Traditional EEG feature extraction methods are often limited to local spatial regions, which restricts their ability to model long-range dependencies across different brain areas. In recent years, the Spatially Adaptive Feature Modulation (SAFM) mechanism has attracted attention for its capacity to weight multi-scale information (Sun et al., 2023). However, when applied to EEG signals, existing SAFM modules still face several challenges: (1) The lack of specificity in the convolution direction can lead to uneven mixing of information. The original SAFM uses a standard 3 × 3 depthwise convolution to model local space. However, EEG signals typically exhibit a clear temporal structure and stable spatial topology, and a large square receptive field may unnecessarily mix information. (2) The scale transformation method does not fully adapt to the temporal characteristics of EEG signals, potentially compromising the integrity of temporal features. Traditional SAFM applies bidirectional pooling across both spatial dimensions when performing multi-scale modeling. Yet, EEG signals demonstrate strong temporal dependencies, and excessive down-sampling may damage these temporal patterns, leading to the loss of crucial temporal features. (3) Excessive scale divisions (*n*_levels). The original SAFM splits features into four scales (*n*_levels = 4), reducing the number of channels per scale, which limits the representation power of each scale. Moreover, frequent transformations between scales can introduce redundant information, undermining the model's discriminative capability.

To address these challenges, we propose the following improvements: (1) Employing 1 × 3 asymmetric convolutions to enhance directional selectivity, which better captures local connection patterns between different brain regions and avoids excessive smoothing. (2) Performing pooling exclusively along the temporal dimension to preserve the temporal flow of EEG signals, while reducing unnecessary computation. (3) Reducing the number of scale divisions by setting *n*_levels to 2, allowing each scale to retain more channels. This enhances the feature representation capacity of individual scales, reduces redundant information, and makes the features more discriminative, ultimately improving the model's ability to differentiate between classes.

**Figure 3:**
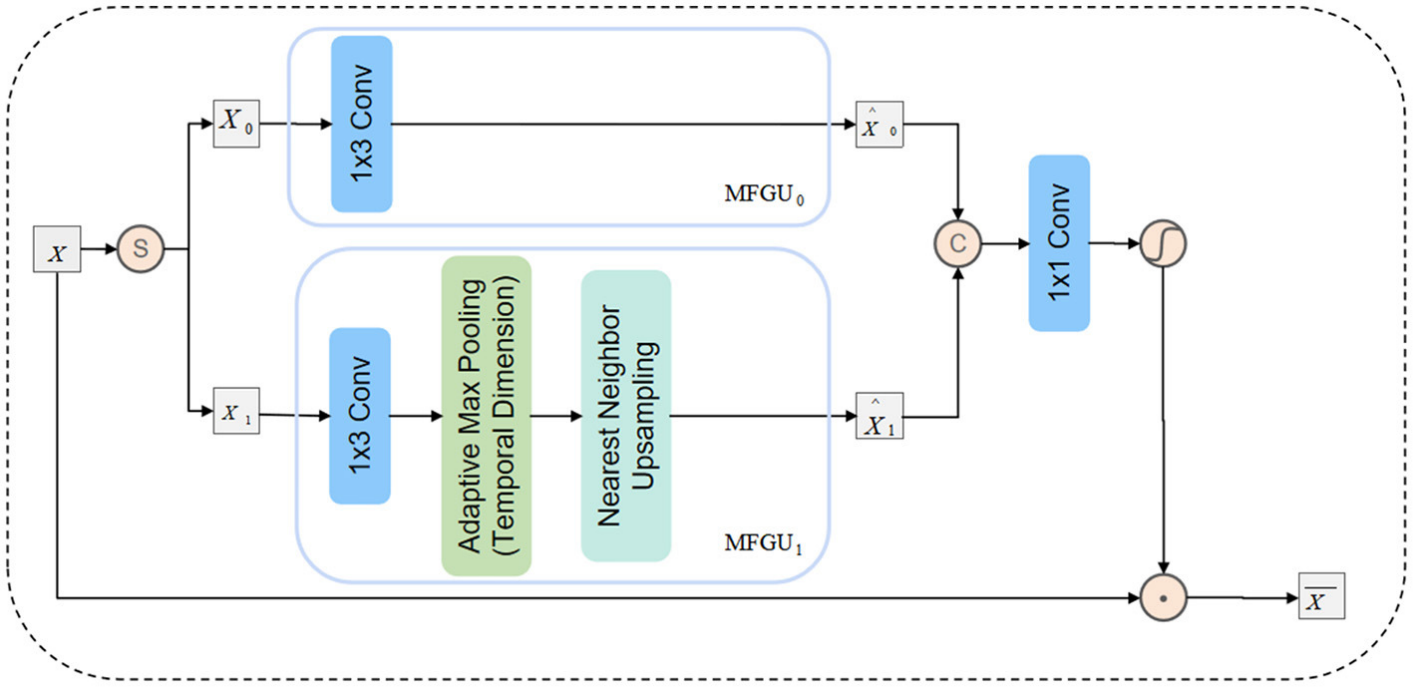
Schematic representation of the internal structure of the directional adaptive feature modulation (DAFM).

As shown in Figure 3, in DAFM, we first split the input feature *X* along the channel dimension into two sub-features [*X*_0_, *X*_1_] and pass them into the Multi-Scale Feature Generation Unit (MFGU). The detailed process is as follows:

First, *X*_0_ is processed through a 1 × 3 depthwise separable convolution to retain high-resolution local information:


(2) $$\begin{array}{l} \left[X_{0},X_{1}\right]=\text{Split}\left(X\right) \end{array}$$

(3) $$\begin{array}{l} {\hat{X}}_{0}={\text{DW-Conv}}_{1\times 3}\left(X_{0}\right) \end{array}$$

Next, *X*_1_ undergoes down-sampling along the temporal dimension through pooling to capture low-frequency information. The down-sampled feature is then processed using depthwise separable convolution, followed by up-sampling to the original resolution through nearest-neighbor interpolation:


(4) $$\begin{array}{l} {\hat{X}}_{1}={↑}_{p}\left({\text{DW-Conv}}_{1\times 3}\left({↓}_{\frac{p}{2}}\left(X_{1}\right)\right)\right) \end{array}$$

where ↑_*p*_(·) represents the nearest-neighbor up-sampling operation, and ${↓}_{\frac{p}{2}}\left(\cdot \right)$ represents down-sampling along the temporal dimension.

Subsequently, the extracted features from different scales are concatenated along the channel dimension and fused through a 1 × 1 convolution to enhance cross-scale information interaction:


(5) $$\begin{array}{l} \hat{X}={\text{Conv}}_{1\times 1}\left(\text{Concat}\left(\left[{\hat{X}}_{0},{\hat{X}}_{0}\right]\right)\right) \end{array}$$

Finally, the fused feature $\hat{X}$ is normalized using the GELU activation function, generating an attention map $\phi \left(\hat{X}\right)$, which is used to adaptively modulate the input feature *X*:


(6) $$\begin{array}{l} \bar{X}=\varphi \left(\hat{X}\right)⊙X \end{array}$$

where φ(·) denotes the GELU activation, and ⊙ denotes element-wise multiplication. Compared with the traditional ReLU function, GELU offers superior performance in nonlinear modeling. Its smooth nonlinear transformation, based on the Gaussian error function, helps alleviate issues such as vanishing or exploding gradients and enhances the model's representational capacity. In this study, we adopt the GELU activation function in the Deep Adaptive Feature Modulation (DAFM) module to apply nonlinear transformation to the fused multi-scale features, thereby generating an adaptive attention map. This attention map enables fine-grained modulation of the input features, enhancing the model's ability to capture discriminative patterns in EEG signals.

#### 3.2.3 CoTAttention module from a dynamic-static perspective

**Figure 4:**
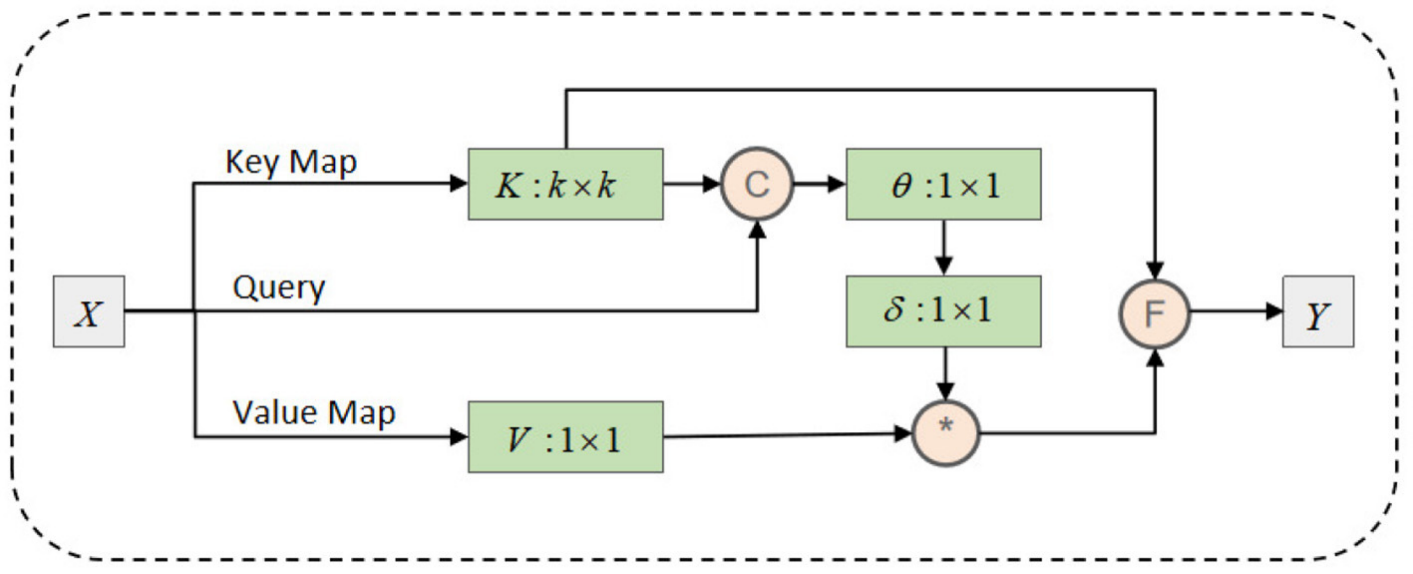
Schematic representation of the internal structure of the CoTAttention module.

As shown in Figure 4, the CoT block begins by applying a group convolution with a kernel size of *k* × *k* to the input feature map *X*, capturing contextual information from the local neighborhood. This step extracts implicit static features from the EEG data, reflecting the fixed spatial relationships between brain regions. For instance, it identifies consistent spatial patterns and relationships between different brain regions, helping to understand fundamental signal patterns within local brain areas.

Next, based on the contextualized key *K*_1_ and query *Q*, the CoT block computes an attention matrix *A* using two consecutive 1 × 1 convolutions. This attention matrix captures the dynamic interactions between brain regions, based on the contextualized key and query features. In EEG data, these dynamic interactions often represent signal variations between brain areas at different time points.

The calculated attention matrix *A* is then used to perform a weighted summation over the values *V*, generating the dynamic context representation *K*_2_. This representation integrates signals from all brain regions, reflecting temporal and spatial signal dynamics, such as synchronization or interaction patterns between brain regions.

Finally, the CoT block fuses the static context representation *K*_1_ with the dynamic context representation *K*_2_ to produce the output *Y*. This fusion combines the fixed spatial patterns of local brain areas with the dynamic interactions of global signals, offering a more comprehensive understanding of the EEG data.

At this stage, the CoTAttention module extracts both static and dynamic features from the high-dimensional EEG information, providing deeper insights into the relationship between brain activity and addiction mechanisms. This analysis not only reveals static brain activity patterns but also captures dynamic changes, offering a thorough perspective on the neural mechanisms underlying addiction.

### 3.3 Stage 3: classifier

As shown in Figure 2, the classifier consists of a global average pooling layer followed by two 1 × 1 convolution layers. The EEG features extracted from the first two stages, including local, global, dynamic, and static features, serve as inputs to the classifier. After passing through the global average pooling layer, each feature map is compressed into a one-dimensional vector, while retaining the global information of the feature maps. This one-dimensional vector is then passed sequentially through the two 1 × 1 convolution layers. Finally, the label with the highest probability in the output represents the network's prediction of whether the EEG data indicates an addiction condition.

### 3.4 Innovations of DSCnet

This study proposes an innovative multi-angle feature learning model, DSCnet, aimed at assisting in the diagnosis of alcohol and drug addiction. The model combines mixed representations, Direction-Adaptive Feature Modulation (DAFM), multi-round feature learning, and a dynamic-static attention mechanism, providing a more precise and efficient solution for EEG signal analysis. The specific innovations are summarized as follows:

Traditional EEG analysis methods, such as PCA, LDA, frequency-domain analysis, and time-frequency analysis, are commonly used to handle high-dimensional EEG data that is noisy and redundant. However, these methods often fail to capture the detailed features of EEG signals comprehensively. In this study, we propose a mixed representation method based on embedding layers and multi-layer convolutions, which effectively extracts various complex features from EEG signals, reduces redundant information, and generates low-dimensional mixed representations. This innovation provides more discriminative features for subsequent alcohol and drug addiction diagnosis, improving diagnostic accuracy.

To fully capture multi-dimensional features in EEG signals, DSCnet adopts a multi-round, multi-angle feature learning strategy. Through depthwise separable convolutions, Direction-Adaptive Feature Modulation (DAFM) modules, and CoTAttention modules, the model deeply explores local, global, dynamic, and static features of EEG signals. This multi-angle learning approach enhances the understanding of the neural mechanisms of alcohol and drug addiction, significantly improving the model's classification performance and generalization ability.

The DAFM module is a key innovation in DSCnet. This module applies 1 × 3 asymmetric convolutions and pooling operations along the time dimension, enabling deep modulation of both the time and spatial features of EEG signals. Additionally, the *n*_levels parameter optimizes the generation of multi-scale features, further enhancing the model's ability to capture long-range dependencies between brain regions in EEG signals. Through these designs, DSCnet can more accurately capture EEG features associated with alcohol and drug addiction, providing strong support for the diagnostic process.

From the dynamic-static perspective, DSCnet introduces the CoTAttention module, which uses grouped convolutions and attention mechanisms to precisely capture static spatial patterns and dynamic time-interaction information in EEG signals. The design of this module allows the model to effectively combine static and dynamic features, comprehensively understanding the time-space interactions in EEG signals, thus improving the recognition of addiction-related neural mechanisms. This innovation not only enhances the model's understanding of brain activity patterns but also provides a novel analysis approach for the auxiliary diagnosis of alcohol and drug addiction.

In conclusion, the DSCnet model, through its innovative mixed representation method, multi-angle feature learning, and dynamic-static attention mechanism, provides a novel diagnostic framework for alcohol and drug addiction EEG signal analysis. These innovations significantly enhance the model's accuracy and robustness in addiction diagnosis and provide in-depth theoretical support and practical guidance for understanding the neural mechanisms of addiction behavior.

## 4 Experiments and results

Our experiments were conducted using Python 3.8 and the PyTorch 2.0.1 framework, with the model trained on an NVIDIA GeForce RTX 4090D 24GB Turbo Edition GPU.

For dataset partitioning, we strictly adhered to the principle of subject independence to ensure the validity of our results. We categorized the data by subjects and allocated 80% of each subject's data to the training set, reserving the remaining 20% for testing. This approach prevents data leakage by ensuring that EEG data from the same subject does not appear in both sets.

We performed multiple binary classification tasks, such as distinguishing between non-alcoholic and alcoholic subjects, as well as non-addicted and addicted individuals. To evaluate the model's performance, we employed several metrics: Accuracy, Precision, Recall, and F1-score, defined as follows:


(7) $$\begin{array}{l} Accuracy=\frac{TP+TN}{TP+TN+FP+FN} \end{array}$$

(8) $$\begin{array}{l} Precision=\frac{TP}{TP+FP} \end{array}$$

(9) $$\begin{array}{l} Recall=\frac{TP}{TP+FN} \end{array}$$

(10) $$\begin{array}{l} F1-score=\frac{2\cdot Precision\cdot Recall}{Precision+Recall} \end{array}$$

Here, *TP*, *TN*, *FP*, and *FN* represent True Positives, True Negatives, False Positives, and False Negatives, respectively.

These metrics collectively demonstrate the effectiveness of our model in binary classification tasks and provide insights into its ability to distinguish between different subject groups.

### 4.1 Comparison with existing algorithms

#### 4.1.1 Comparison with machine learning models

In the field of EEG classification, machine learning is widely employed due to its strong interpretability and effectiveness; however, it heavily depends on manual feature extraction and domain expertise. In contrast, deep learning models can automatically extract features without human intervention. In this study, we employed the following versions of machine learning models for comparison: For AdaBoost, we used 400 base estimators with the SAMME algorithm. The Bagging model was configured with 300 base estimators using BaggingClassifier. The Extra Trees model used ExtraTreesClassifier with 2,000 base estimators. Gradient Boosting was implemented with 300 base estimators and a learning rate of 0.1 using GradientBoostingClassifier. The Random Forest model utilized 350 base estimators in RandomForestClassifier. For Stacking, we used RandomForestClassifier, GradientBoostingClassifier, and SVC, each with 100 base estimators, as base learners, and LogisticRegression as the final meta-learner. The Support Vector Classifier (SVC) was configured with a radial basis function (RBF) kernel, with the *C* parameter set to 10 and gamma set to “scale.” The Voting classifier adopted a soft voting strategy, incorporating a logistic regression model (LogisticRegression), a random forest classifier (RandomForestClassifier with 50 trees), and a support vector classifier (SVC with probability estimation enabled). As shown in Table 1, our model consistently outperforms all others across various metrics in both the drug addiction and alcohol addiction datasets.

**Table 1:** Comparison of DSCnet with classical machine learning algorithms.

	**Drug addiction dataset**				**Alcohol addiction dataset**			
**Methods**	**Accuracy (%)**	**Precision (%)**	**Recall (%)**	**F1-score (%)**	**Accuracy (%)**	**Precision (%)**	**Recall (%)**	**F1-score (%)**
AdaBoost	65.95	66.09	65.95	65.62	79.36	78.98	79.36	78.82
Bagging	74.68	77.23	74.68	73.78	81.96	81.77	81.96	81.82
ExtraTrees	71.87	78.25	71.86	69.67	80.32	79.98	80.32	79.95
GradientBoosting	73.17	74.65	73.17	72.50	80.58	81.26	80.59	79.33
RandomForest	73.13	77.44	73.13	71.64	79.36	79.09	79.36	79.17
Stacking	72.50	76.13	72.50	71.11	82.97	82.74	82.97	82.68
SVC	55.95	56.61	55.95	52.28	76.71	76.15	76.71	76.00
Voting	54.36	54.09	54.37	53.73	83.06	83.06	83.06	83.06
DSCnet	85.11	85.13	85.12	85.12	84.56	84.73	84.56	84.63

Notably, the performance of the SVC and Voting classifiers on the drug addiction dataset is significantly lower than on the alcohol addiction dataset, with a performance gap of 20% to 30%. For the other machine learning models, the difference is around 10%. This discrepancy may be attributed to the fact that the features in the drug addiction EEG data are more subtle and challenging for traditional models to capture. Therefore, our model not only demonstrates exceptional classification performance but also addresses the limitations of machine learning models in effectively learning features from drug addiction data.

#### 4.1.2 Comparison with deep learning models

Deep learning has become widely adopted in the classification and diagnosis of EEG signals. For comparison, we selected three classical deep learning models–Convolutional Neural Network (CNN), Long Short-Term Memory (LSTM), and Transformer Encoder Classifier (TEC)–as well as models commonly used in multivariate time series classification, including DSN (Xiao et al., 2022), Mgformer (Wen et al., 2024), and EEG Conformer (EEG-C) (Song et al., 2022), which is specifically designed for EEG classification. Among these, the CNN model consists of two 1D convolutional layers (with a kernel size of 7), followed by ReLU activation and batch normalization, and concludes with a fully connected layer after flattening. Xavier initialization is applied to all convolutional and linear layers. The LSTM model is composed of two stacked LSTM layers and a fully connected layer, with He, orthogonal, and Xavier initialization strategies. The Transformer Encoder Classifier (TEC) model is built with two layers of single-head Transformer encoders and a fully connected layer, utilizing global average pooling to extract time-domain features. These models were evaluated on our datasets, and the results are summarized in Table 2. In comparison to the machine learning models presented in Table 1, deep learning models demonstrated superior overall performance.

**Table 2:** Comparison of DSCnet with other deep learning algorithms.

	**Drug addiction dataset**				**Alcohol addiction dataset**			
**Methods**	**Accuracy (%)**	**Precision (%)**	**Recall (%)**	**F1-score (%)**	**Accuracy (%)**	**Precision (%)**	**Recall (%)**	**F1-score (%)**
CNN	80.27	80.55	80.27	80.16	81.96	81.77	81.96	81.83
LSTM	73.45	73.84	73.45	73.19	70.05	70.06	70.05	70.05
TEC	74.48	74.58	74.48	74.50	79.09	79.31	79.09	79.18
DSN	65.63	65.82	65.63	65.24	79.00	79.08	79.00	77.81
Mgformer	77.58	77.60	77.58	77.58	81.87	81.89	81.87	81.88
EEG-C	79.84	80.71	79.84	79.58	80.73	80.95	80.73	80.82
DSCnet	85.11	85.13	85.12	85.12	84.56	84.73	84.56	84.63

A noteworthy observation is that machine learning models exhibited significant performance imbalances between the drug addiction and alcohol addiction datasets. In contrast, deep learning models showed much more stability across both datasets, significantly reducing this imbalance.

Among the deep learning models compared, CNN outperformed both LSTM and TEC in terms of performance and balance. This advantage may be attributed to CNN's ability to effectively capture the spatio-temporal patterns inherent in EEG signals. Furthermore, our proposed DSCnet surpassed all other deep learning models, demonstrating superior performance and enhanced balance across both datasets. This underscores the effectiveness of DSCnet in learning complex EEG patterns for addiction diagnosis.

#### 4.1.3 Comparison with existing methods for alcohol addiction

We conducted a comprehensive comparison of our proposed model with recent alcohol addiction diagnosis models. Hu et al. (2020) introduced an innovative linear discriminant analysis (LDA) method that combines an effective nuclear norm penalty to enhance the application of low-rank structures in alcohol addiction classification. In addition to their proposed model, Hu et al. conducted several experiments, including: (1) Logistic Lasso: a regularized matrix logistic regression classification method using Lasso penalty; (2) Logistic Nuclear: a regularized matrix logistic regression classification method with nuclear norm penalty; and (3) Lasso LDA: a classification method integrating the naive Lasso penalty within the LDA framework. Aarthi et al. (2023) proposed a deep learning model based on autoencoders and bidirectional long short-term memory networks (Bi-LSTM), using ReLU and Sigmoid activation functions (applied to the Dense layer and output layer, respectively) to predict alcohol addiction from EEG signals. Rizal et al. (2023) proposed a feature extraction technique based on Gray-Level Co-occurrence Matrix (GLCM) texture analysis combined with random forest classification for alcohol addiction detection. Sedrati et al. (2023) proposed a model based on the Discrete-to-Continuous (DtC) algorithm, which reduces dataset dimensionality by selecting the most relevant EEG channels, using time-domain features and logistic regression (LR) for binary classification, and evaluating the effectiveness of the DtC algorithm in alcohol use disorder (AUD) detection. Li and Xiao (2023) proposed a latent function factor model that introduces dependencies between different functions via unobserved stochastic processes. Min et al. (2023) provided a minimax lower bound for the estimation and prediction errors of a tensor discriminant analysis (HD-TDA) model, comparing these methods with commonly used sparse discriminant analysis methods for vector data, such as L1-FDA and MSDA, while also involving pioneering matrix and tensor discriminant analysis methods, including PLMC, CMDA, and STDA. Aprillia et al. (2024) proposed a feature extraction model based on texture analysis, treating EEG signals as matrices with N channels and M samples, normalizing them into 8-bit images, then extracting five features using the Gray-Level Difference Matrix (GLDM) method and classifying them using linear discriminant analysis (LDA). Huang et al. (2024) introduced the RMRE model, a matrix covariance regression model based on low-rank constraints and additional regularization terms for analyzing structured signals, and compared it with low-rank estimation matrix regression estimators (LEME), SDNCMV, and spectral regularization regression estimators (SRRE) for alcohol classification tasks. Buriro et al. (2025) proposed WideConvNet, an improved convolutional neural network (CNN) that incorporates a modified inception module designed specifically for 1D data, utilizing filters of varying sizes to capture temporal patterns in EEG signals, thereby effectively classifying event-related potentials (ERP).

Given that many existing studies primarily utilize accuracy as the main evaluation metric for classification, we adopted this metric for our comparative analysis as well. The results of this comparison are summarized in Table 3, demonstrating that our proposed method significantly outperforms other models in terms of accuracy, with improvements ranging from 0.16% to 18.39%. These findings highlight the superior reliability of our model in diagnosing alcohol addiction, emphasizing its effectiveness and advantages in practical applications. This indicates that DSCnet not only enhances classification accuracy but also holds promise for providing more accurate and reliable assessments in clinical settings for alcohol addiction.

**Table 3:** Comparison of DSCnet with other advanced algorithms on the alcohol addiction dataset.

**References**	**Method**	**Accuracy (%)**
Hu et al. (2020)	Logistic Lasso	73.76
	Logistic Nuclear	75.56
	Lasso LDA	75.88
	LDA+nuclear norm penalized regression	77.80
Aarthi et al. (2023)	Auto-Encoder	65.50
Rizal et al. (2023)	GLCM + Random Forest	70.00
Sedrati et al. (2023)	DtC-selected EEG channels (C3, CP5, PO7, F8)	82.60
	DtC-Irrelevant Channels (AF8, CZ, FC5, FT7)	69.56
Li and Xiao (2023)	Latent factor model	84.40
Min et al. (2023)	STDA	66.17
	PLMC	67.75
	CMDA	68.50
	l1-FDA	73.75
	MSDA	74.67
	HD-TDA	77.83
Aprillia et al. (2024)	GLDM + LDA	73.30
Huang et al. (2024)	LEME	76.00
	SDNCMV	77.00
	SRRE	79.00
	RMRE	79.00
Buriro et al. (2025)	WideConvNet	68.00
Our	DSCnet	84.56

### 4.2 Ablation study

#### 4.2.1 Effectiveness analysis of the embedding layer

The primary function of the embedding layer is to construct a low-dimensional representation of the original EEG, eliminating noise and redundancy while maintaining connectivity to the original EEG in the channel dimension, thus forming a Hybrid Representation of EEG. To evaluate the performance of the embedding layer, we compared models with and without it, as shown in Table 4. The results indicate that our method significantly outperforms the model without the embedding layer across all metrics on the dataset. This demonstrates that constructing a low-dimensional representation of the EEG through the embedding layer helps mitigate the heterogeneity of redundant features. Therefore, directly inputting the original EEG into the model for multi-angle feature extraction does not effectively distinguish between addicted and non-addicted patients.

**Table 4:** Effect of the embedding layer on DSCnet.

	**Drug addiction dataset**				**Alcohol addiction dataset**			
**Methods**	**Accuracy (%)**	**Precision (%)**	**Recall (%)**	**F1-score (%)**	**Accuracy (%)**	**Precision (%)**	**Recall (%)**	**F1-score (%)**
w/o embedding	75.99	77.48	75.99	75.45	76.89	76.45	76.89	76.56
w embedding	85.11	85.13	85.12	85.12	84.56	84.73	84.56	84.63

#### 4.2.2 Effectiveness analysis of depthwise separable convolution

To validate the effectiveness of depthwise separable convolution (DSC) in the model, we replaced DSC with standard convolution (SC) while keeping the input and output dimensions unchanged and maintaining other parameters as consistent as possible for comparison. The experimental results are shown in Table 5. In the drug addiction dataset, compared to the control group, the model using depthwise separable convolution demonstrated significant advantages across various metrics, with accuracy increasing by 4.28%, precision by 3.66%, recall by 4.29%, and F1-score by 4.48%. In the alcohol addiction dataset, this model also exhibited superior performance, with accuracy increasing by 2.84%, precision by 2.8%, recall by 2.79%, and F1-score by 2.79%. These results indicate that depthwise separable convolution outperforms standard convolution in capturing local information related to addiction mechanisms within EEG data.

**Table 5:** Effect of depthwise separable convolution on DSCnet.

	**Drug addiction dataset**				**Alcohol addiction dataset**			
**Methods**	**Accuracy (%)**	**Precision (%)**	**Recall (%)**	**F1-score (%)**	**Accuracy (%)**	**Precision (%)**	**Recall (%)**	**F1-score (%)**
SC	80.83	81.47	80.83	80.64	81.78	81.93	81.78	81.85
DSC	85.11	85.13	85.12	85.12	84.57	84.73	84.57	84.63

#### 4.2.3 Effectiveness analysis of the DAFM

Relying solely on a local perspective to capture addiction mechanisms in EEG data results in limited feature extraction. To address this, we designed the DAFM module, which adopts a global perspective to comprehensively capture EEG features. The design of the DAFM module is inspired by the SAFM module. However, due to incompatible input and output dimensions, the SAFM module cannot be directly applied to our model and, therefore, cannot be directly compared. To evaluate the effectiveness of the DAFM module and the choice of *n*_level, we compared it with a control group that does not include the DAFM module, as well as with the DAFM module set to *n*_level = 4. The experimental results, as shown in Table 6, demonstrate that in both the drug addiction and alcohol addiction datasets, models using the DAFM module–whether set to *n*_level = 4 or *n*_level = 2–outperform those without the DAFM module across all evaluation metrics. Furthermore, the model with *n*_level = 2 performs better than the one with *n*_level = 4. Additionally, we observed that in the alcohol addiction dataset, the impact of using the DAFM module with *n*_level = 2 on the evaluation metrics is more pronounced. This may suggest that, compared to drug addiction, capturing features related to alcohol addiction requires a more global perspective. However, this hypothesis needs further research and validation.

**Table 6:** Effect of the DAFM on DSCnet.

	**Drug addiction dataset**				**Alcohol addiction dataset**			
**Methods**	**Accuracy (%)**	**Precision (%)**	**Recall (%)**	**F1-score (%)**	**Accuracy (%)**	**Precision (%)**	**Recall (%)**	**F1-score (%)**
w/o DAFM	81.15	81.15	81.15	81.14	75.94	75.31	75.94	75.11
w DAFM (*n*_levels = 4)	83.69	83.70	83.69	83.67	79.00	78.61	79.00	78.36
w DAFM (*n*_levels = 2)	85.11	85.13	85.12	85.12	84.57	84.73	84.57	84.63

#### 4.2.4 Effectiveness analysis of the CoT module

Previous research has primarily focused on analyzing local and global features within EEG signals, often neglecting the rich dynamic and static characteristics inherent in the data. This oversight is significant, as it may result in a critical dimension of information being overlooked, which is essential for effectively distinguishing between healthy individuals and those suffering from addiction. Recognizing the importance of these features, we aimed to investigate whether the incorporation of the Coherent Transformation (CoT) module in the DSCnet model could enhance the differentiation of dynamic and static features, ultimately improving classification accuracy.

To explore this hypothesis, we conducted a series of experiments designed to assess the impact of the CoT module on classification performance. The results of these experiments, detailed in Table 7, indicate a notable improvement in classification outcomes for both alcohol addiction and drug addiction across all evaluation metrics. These findings strongly suggest that the EEG signals of individuals with addiction exhibit discernible differences compared to those of healthy individuals, particularly in terms of their dynamic and static features. By integrating the CoT module, our model appears to capture these distinctions more effectively, highlighting the potential of advanced feature extraction techniques in enhancing our understanding of addiction-related neural patterns. This advancement not only contributes to the field of EEG analysis but also has implications for the development of more accurate diagnostic tools for addiction.

**Table 7:** Effect of the CoT Module on DSCnet.

	**Drug addiction dataset**				**Alcohol addiction dataset**			
**Methods**	**Accuracy (%)**	**Precision (%)**	**Recall (%)**	**F1-score (%)**	**Accuracy (%)**	**Precision (%)**	**Recall (%)**	**F1-score (%)**
w/o CoT module	80.08	80.74	80.08	79.87	79.91	79.57	79.91	79.60
w CoT module	85.11	85.13	85.12	85.12	84.57	84.73	84.57	84.63

#### 4.2.5 Effectiveness analysis of feature extraction count

In terms of feature extraction, past research typically selects either one or three or more rounds of feature extraction. However, in our model, we chose to perform feature extraction twice in the second stage. The results in Table 8 show that models performing only one round of feature extraction had lower metrics compared to those performing it twice, as a single extraction may not sufficiently capture the key information in the data. Additionally, we found that when three rounds of feature extraction were conducted, the model's metrics were actually lower than those with two rounds. This may be due to excessive feature extraction leading to overly abstract features, which can negatively affect the model's final classification performance.

**Table 8:** Effect of feature extraction count in the second stage of DSCnet.

	**Drug addiction dataset**				**Alcohol addiction dataset**			
**Methods**	**Accuracy (%)**	**Precision (%)**	**Recall (%)**	**F1-score (%)**	**Accuracy (%)**	**Precision (%)**	**Recall (%)**	**F1-score (%)**
One	80.08	82.60	80.08	79.50	81.32	81.03	81.32	81.03
Two	85.11	85.13	85.12	85.12	84.57	84.73	84.57	84.63
Three	80.83	80.85	80.83	80.83	76.26	76.70	76.26	76.43

## 5 Discussion

Alcohol addiction is a progressive and chronic relapsing disease that can lead to various neurological disorders, resulting in severe health consequences (Yunusoğlu, 2021, 2022). In recent years, EEG analysis has been widely applied to the study and diagnosis of addiction mechanisms. Previous methods mainly involved extracting features after obtaining low-dimensional representations of EEG data, with traditional methods often utilizing Principal Component Analysis (PCA) and Independent Component Analysis (ICA). Based on this, we introduced a convolutional embedding layer to construct low-dimensional representations of EEG while employing skip connections to generate a Hybrid Representation that highlights key features of EEG while retaining other characteristics. We further conducted feature learning from multiple perspectives–local, global, dynamic, and static–and ultimately deeply fused the extracted feature matrices for addiction diagnosis.

To our knowledge, the constructed drug addiction dataset is the largest EEG addiction dataset currently available, and our model is the first to assist in the simultaneous diagnosis of both alcohol and drug addiction. Experimental results show that DSCnet outperforms other classical algorithms on both the alcohol addiction dataset and the drug addiction dataset we constructed. Our approach provides a new perspective for analyzing addiction features in EEG, opening new research avenues for other researchers.

Despite the promising performance of our model in diagnosing drug and alcohol addiction, it has several limitations:

- Our model has only been validated on resting-state drug addiction data, which may influence its effectiveness in other contexts. Future work will involve collecting task-state data from participants to train the model across diverse modalities, enhancing its applicability.
- The current evaluation of the model is restricted to datasets related to alcohol and drug addiction, with no assessment of its performance on other brain disorders. In future studies, we intend to integrate datasets from various neurological conditions to validate the model's versatility and expand its potential for broader applications.

## 6 Conclusions

This paper constructs a drug addiction dataset that encompasses a broader group of participants and proposes a multi-angle feature learning model, DSCnet, based on a Hybrid Representation of EEG. Compared to previous datasets, this drug addiction dataset provides a richer foundation for validating the generalization capabilities of the model. DSCnet extracts low-dimensional EEG features through embedding methods and integrates the original EEG signals with low-dimensional features using skip connections to construct a Hybrid Representation. In the feature learning process, DSCnet employs different modules to extract local, global, dynamic, and static features of EEG. Among them, we independently designed the Directional Adaptive Feature Modulation (DAFM) module for global feature extraction. DAFM adaptively adjusts the directionality of features, effectively capturing global EEG information. This module enhances feature representation while preserving critical spatial-temporal information, allowing the model to more comprehensively capture neural activity patterns associated with addiction mechanisms. Furthermore, DSCnet performs deep fusion of high-order features and is ultimately applied to the auxiliary diagnosis of drug and alcohol addiction mechanisms. Experimental results demonstrate that DSCnet achieves the best performance across multiple metrics and effectively addresses the issue of imbalanced accuracy found in other models on both datasets, confirming its validity.

## Data Availability

The original contributions presented in the study are included in the article/supplementary material, further inquiries can be directed to the corresponding authors.
